# Are Algae Relevant to the Detritus-Based Food Web in Tank-Bromeliads?

**DOI:** 10.1371/journal.pone.0020129

**Published:** 2011-05-18

**Authors:** Olivier Brouard, Anne-Hélène Le Jeune, Céline Leroy, Régis Cereghino, Olivier Roux, Laurent Pelozuelo, Alain Dejean, Bruno Corbara, Jean-François Carrias

**Affiliations:** 1 LMGE, Laboratoire Microorganismes: Génome et Environnement, Université Blaise Pascal, UMR CNRS 6023, Aubière, France; 2 Ecologie des Forêts de Guyane, UMR CNRS 8172, Campus Agronomique, Kourou, France; 3 EcoLab, Laboratoire d'Ecologie Fonctionnelle et Environnement, Université Paul Sabatier, UMR CNRS 5245, Toulouse, France; US Dept. of Agriculture – Agricultural Research Service (USDA-ARS), United States of America

## Abstract

We assessed the occurrence of algae in five species of tank-bromeliads found in contrasting environmental sites in a Neotropical, primary rainforest around the Nouragues Research Station, French Guiana. The distributions of both algal abundance and biomass were examined based on physical parameters, the morphological characteristics of bromeliad species and with regard to the structure of other aquatic microbial communities held in the tanks. Algae were retrieved in all of the bromeliad species with mean densities ranging from ∼10^2^ to 10^4^ cells/mL. Their biomass was positively correlated to light exposure and bacterial biomass. Algae represented a tiny component of the detrital food web in shaded bromeliads but accounted for up to 30 percent of the living microbial carbon in the tanks of *Catopsis berteroniana*, located in a highly exposed area. Thus, while nutrient supplies are believed to originate from wind-borne particles and trapped insects (*i.e.*, allochtonous organic matter), our results indicate that primary producers (*i.e.*, autochtonous organic matter) are present in this insectivorous bromeliad. Using a 24-h incubation of size-fractionated and manipulated samples from this plant, we evaluated the impact of mosquito foraging on algae, other microorganisms and rotifers. The prey assemblages were greatly altered by the predation of mosquito larvae. Grazing losses indicated that the dominant algal taxon, *Bumilleriopsis* sp., like protozoa and rotifers, is a significant part of the diet of mosquito larvae. We conclude that algae are a relevant functional community of the aquatic food web in *C. berteroniana* and might form the basis of a complementary non-detrital food web.

## Introduction

Bromeliads (Bromeliaceae) are common flowering plants in the Neotropics [1]. The majority of bromeliad species have tightly-interlocking leaves that impound water in a central cup, and leaf axils forming a phytotelm (“plant-held water”). About half of the phytotelm plants in tropical America are tank-bromeliads [2], and their collective diversity and density form a major fragmented aquatic ecosystem that provides habitat, breeding space and food for diverse organisms [1], [3]. They are, for example, major development sites for aquatic invertebrates [1], and their associated aquatic biota form valuable model systems for food web studies by combining the advantages of field-based studies and microcosms [3], [4]. The watertight cavities of tank-bromeliads trap leaf litter and wind-borne particles that constitute a source of nutrients for both the aquatic food web and the bromeliad [5]. Dead organisms, particulate organic matter (POM) and faecal particles collect in the bases of the leaves and are utilized by bacteria and other microorganisms which are then preyed upon by larger invertebrates [6], [7]. Surveys on the aquatic food webs of tank-bromeliads have mainly focused on macro-organisms, especially mosquito larvae [2], [7], [8], leaving the microbial communities under-studied [9], [10].

Most of our current knowledge on the microbial communities inhabiting phytotelmata comes from studies conducted on the North American pitcher plant *Sarracenia purpurea* [11]–[16]. Bacteria form the first trophic level exploiting small organic matter and nutrients. Protozoa and rotifers feeding on bacteria constitute the first predatory level. This detrital microbial food web is largely controlled by filtering mosquito larvae [11], [12] and lacks primary producers [12], [16]. The earliest limnological studies of tank-bromeliads reported the presence of algae [6], [17], although without providing quantitative data. Nevertheless, algae seem to be scarce in the pitchers of *S. purpurea* populations in North America. A significant algal community was, however, found growing in the pitchers of an allochtonous population of *S. purpurea* studied in Europe [18], suggesting that algae might constitute an important food source for the predators and an indirect nutrient supply for the plant. The occurrence of phototrophic microorganisms in bromeliad ecosystems raises the question of the contribution of algae to the organic carbon sources for the food web.

Although it is generally accepted that bromeliaceous food webs rely on heterotrophic microbial pathways, we hypothesized that the occurrence of algae would increase with the greater exposure of the plants to sunlight, leading to the existence of an autotrophic pathway. Because of the wide diversity of habitats colonized by tank-bromeliads in the primary eastern Amazonian rainforest, we investigated the relative contribution of algae to the tank-bromeliad food web in contrasting sites differing in elevation and forest structure. Particular attention was given to the presence of algae within the microbial communities and based on the position of the foliar chambers, the plant species, and changes in abiotic factors. We also conducted experiments to evaluate the importance of metazoan mosquito larvae in the top-down control of algae and other groups of organisms inhabiting the most exposed plants.

## Methods

### Ethics Statement

This study was conducted according to relevant national and international guidelines.

### Study site

**Figure 1:**
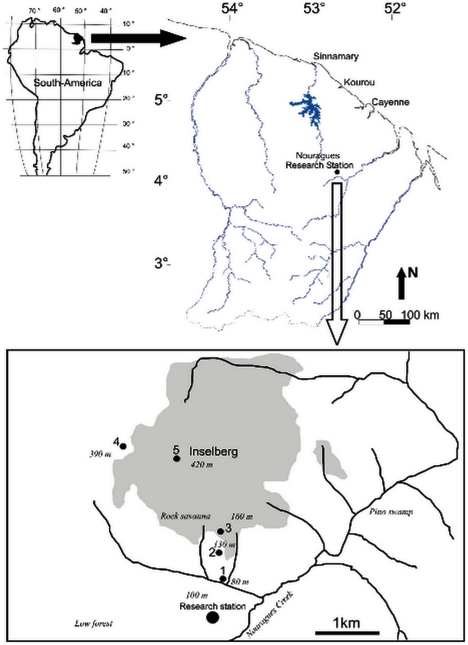
Map of the study area and location of the four sampling stations (1–4). The grey area delineates the inselberg site (maximum elevation = 430 m asl).

The study was conducted in a primary rainforest characteristic of the eastern Amazon, around the Nouragues Tropical Forest Research Station (4°5′N, 52°41′W, French Guiana). The area is totally uninhabited, and anthropogenic disturbance is almost nil. The Nouragues Station is located in the Nouragues Natural Reserve (Fig. 1) 100 km from Cayenne and 40 km from the nearest village (Regina). The area is delineated by hills (elevation <120 m asl) and by the Balenfois Mountains (maximum elevation: 460 m asl). A granite inselberg dominates the Nouragues Station (maximum elevation: 420 m asl). The vegetation is composed of primary rainforest with small, naturally-occurring stands of palm forest on poorly-drained terrain (“pino swamps”), liana forests, and bamboo thickets. On the inselberg, relictual patches of savanna (“rock-savannas”) can be found intermingled with patches of shrubby trees belonging to the Clusiaceae, Myrtaceaea and Bombacaceae families [19]. The climate is tropical moist, with 3,000 mm of annual precipitation distributed over 280 days. There is a major drop in rainfall between September and November (the dry season) and another shorter and more irregular dry period in March. The maximum and minimum monthly temperatures average 33.5°C (32.1–35.8°C) and 20.3°C (19.7–21°C), respectively.

### Sampling and field data

**Figure 2:**
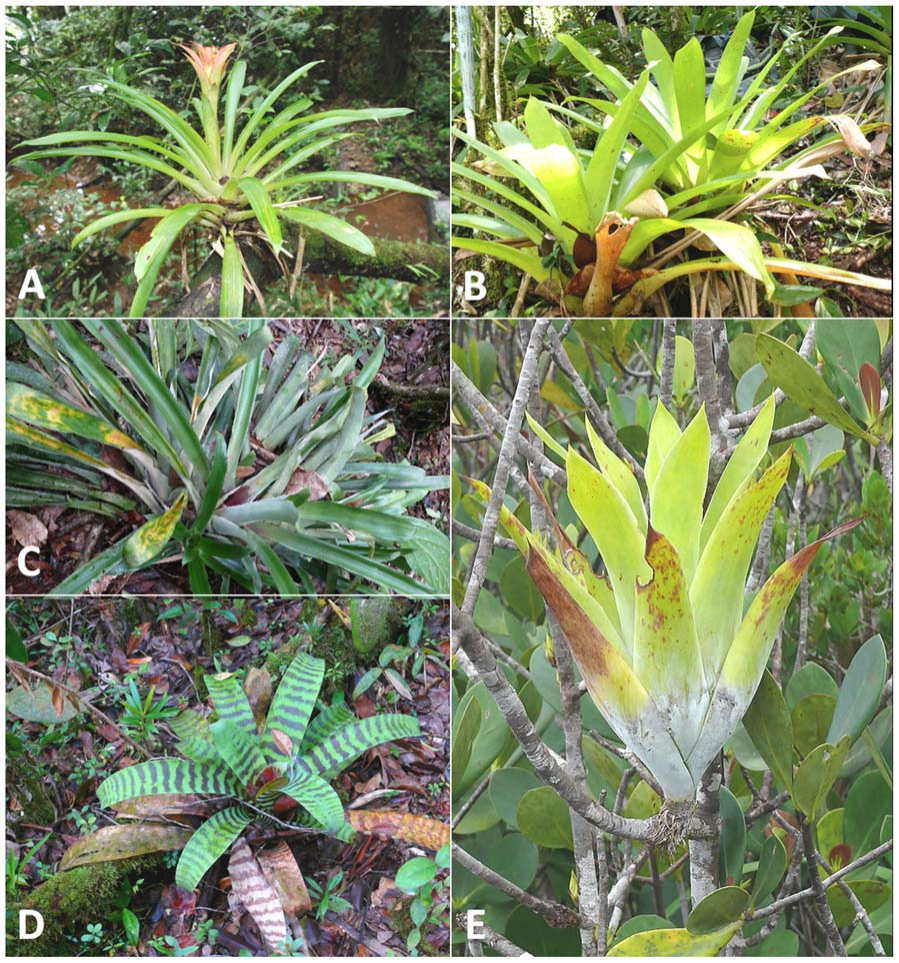
The five species of tank-bromeliads sampled during this study (A = *Guzmania lingulata*, B = *Vriesea pleiosticha*, C = *Aechmea bromeliifolia*, D = *Vriesea splendens*, E = *Catopsis berteroniana*).

Field data acquisition and sampling was carried out during the April 2008 rainy season. We selected five bromeliad species located in four different sampling stations at two sites around the Nouragues Station (Table 1). Sample collections necessary to scientific research are authorized by the Nouragues Field Station Scientific Board, provided that their impact upon the environment is considered negligible (see guidelines at http://www.nouragues.cnrs.fr/GB-collections.html). In accordance with these guidelines, we used a non-destructive sampling technique to extract the water contained in the plants (i.e. the bromeliads were not harvested, nor dismantled - see sampling technique below). *Guzmania lingulata* (L.) Mez, *Vriesea pleiosticha* (Grisebach) Gouda and *Aechmea bromeliifolia* (Rudge) Baker were situated in a transitional forest at 130 m asl while *Vriesea splendens* (Brongniart) Lemaire was situated in a forested area on the inselberg at 390 m asl (Table 1; Fig. 2). *Catopsis berteroniana* (Schultes f.) Mez was restricted to the summit of the inselberg (420 m asl, above the tree-line), where it was an epiphyte on *Clusia minor* shrubs. We only considered mature bromeliads at the end of the plant lifecycle flowering stage to avoid bias from ontogenetic gradients in our analyses.

**Table 1:** Main characteristics of the five bromeliad species sampled during the study at the Nouragues station (French Guiana) in April 2008.

Sites	Transitional forest			Inselberg	
Stations	N° 1: Shaded forest	N° 2: Tree gap		N° 3: Forested area	N° 4: Open area
Elevation (m)	130	130		390	420
Light (%)***	15.8±2.8**^a^**	24.2±3.1**^b^**		16.3±3.4**^a^**	72.5±13.3**^c^**
Species	*G. lingulata*	*V. pleiosticha*	*A. bromeliifolia*	*V. splendens*	*C. berteroniana* δ
Life form	Epiphytic & epilithic	Epiphytic & epilithic	Epiphytic & epilithic	Epiphytic & terrestrial	Epiphytic
Reservoir					
Diameter (cm)**	2.8±0.5**^c^**	11.1±3.9**^ab^**	5.8±1.1**^a^**	11.3±0.6**^bd^**	2.7±0.3**^cd^**
Height (cm)*	7.3±0.9**^c^**	19.2±8.5**^abc^**	12.9±3.4**^b^**	17.0±1**^bc^**	7.0±1.5**^ac^**
pH					
Central pool*	5.9±0.1**^ac^**	4.9±0.4**^b^**	5.3±0.2**^bc^**	5.5±0.3**^abc^**	6.3±0.7**^c^**
Outer axils	5.4±0.5**^a^**	5.3±0.2**^a^**	5.4±0.2**^a^**	5.3±0.3**^a^**	5.5±0.8**^a^**
T (°C)					
Central pool*	26.4±0.5**^ac^**	24.3±0.5**^b^**	25.6±0.4**^a^**	23.1±0.2**^c^**	25.1±0.9**^abc^**
Outer axils***	26.0±0.3**^a^**	24.1±0.5**^b^**	25.9±0.2**^a^**	23.1±0.2**^c^**	25.5±1.2**^abc^**

δ insectivorous taxon.

Asterisks indicate significant differences after the Kruskal-Wallis test (*P<0.05, **P<0.01, ***P<0.001). Values marked with the same letter are not significantly different (Mann-Whitney pairwise comparisons, *P*>0.05). For pH and temperature differences refer to plant species.

The intensity of transmitted light on the bromeliads was evaluated using hemispherical photography. Photographs were taken near dusk to avoid direct sunlight, and from three to four positions for each bromeliad patch. A fisheye converter lens (Nikon, FC-E8 0.21×) mounted on a digital camera (Nikon Coolpix 4500) was used to provide a 180° canopy view. Digital images were analyzed with Gap Light Analyzer (GLA) 2.0 image processing software to calculate the percentage of total incident radiation [20].

As the bromeliads could not be removed from their host trees for legal reasons (the sites are located in a protected area), the aquatic communities were sampled by collecting the water retained in the tanks. We sampled a total of 55 tanks from 22 different plants belonging to five bromeliad species. For each plant (three to five individuals from each species), the central pool and one or two tanks in the outer axils were sampled. Prior to sampling, temperature (°C) and pH were measured in each tank using an EcoScan meter and probe (Eutech Instruments Pte Ltd/Oakton Instruments). Reservoir height and diameter (two random measurements at 90°) were recorded. Water samples were collected in a 5-mL automatic pipette, filtered through a 150-µm pore-size nylon screen, and immediately fixed with 4 percent (final concentration) formaldehyde in 15-mL plastic tubes (Falcon®). Sample collection, temperature and pH measurements were carried out from 11 h to 14 h to reduce daily variability.

### Microbial Communities' structures

Algae and heterotrophic nanoflagellates (HNF) were counted using epifluorescence microcopy. Subsamples of 1–5 mL were stained with primulin [21], collected onto 0.8 µm pore-size black Nuclepore filters, mounted with oil between a glass slide and a cover slip, and then stored at −20°C until counting was performed. The slides were examined under UV light (340–380 nm) at ×1100 magnification on a Leica DC 300F epifluorescence microscope. Pigmented protists were detected using the red autofluorescence of chlorophyll *a* under blue light (450–490 nm) excitation. Heterotrophic bacteria were counted using a FACSCalibur flow cytometer (Becton Dickinson). One mL subsamples were filtered through a 10 µm pore-size nylon screen to remove the largest organic particles which can obstruct the cytometer. Five µL of the filtrate were then transferred to tubes appropriate for flow cytometry (Falcon®, dimension 12×75 mm), diluted in 445 µL (0.02 µm filtered) TE buffer (20 mM Tris-Cl and 2 mM EDTA, pH 8) and incubated with 5 µL SYBR Green I (10^−2^ dilution of the commercial stock solution; Molecular Probes) for 15 minutes. Subsamples of 2–10 mL were placed in plankton chambers to count ciliated protozoa. Counting was conducted using an inverted Leitz Laborlux microscope equipped with an image analyzer at ×400 magnification by scanning the entire chamber. Ciliates were classified according to dimensions and body shape.

The biomasses of microbial communities were estimated from the mean volume of bacteria and each algal and protozoan taxon based on the corresponding geometric shapes. A mean bacterial biovolume of 1.6 µm^3^ was calculated through the microscopic analysis of a total of 20 samples. The total volume of algae, flagellates and ciliates was calculated by summing up the individual volume of each taxon. Carbon content was then estimated using carbon-volume conversion factors. We considered that 10^6^ µm^3^ = 1 µg (wet weight) and assumed that organic cell carbon represents 12 percent of the wet weight biomass for algae [22]. The carbon content of other microbial communities was calculated assuming 204 fgC µm^−3^ for bacteria [23], 200 fgC µm^−3^ for flagellates [24] and 190 fgC µm^−3^ for ciliates [25].

### Impact of mosquito larvae on aquatic communities

The fluid from the tanks of several *Catopsis berteroniana* was pooled into a large plastic container for a total volume of 600 mL, and kept at an ambient temperature during 24 h at the Nouragues Station before the start of the experiment. The entire volume was then filtered through a 150-µm pore-size nylon screen to remove the largest organic particles and collect large metazoans. Control tests indicated that rotifers and microorganisms (e.g. bacteria, HNF, ciliates and microalgae) were not removed by filtration through the 150-µm screen, and were thus all present in the water for subsequent experiments. Larvae were collected from various bromeliads near the field station, and then sorted and identified to morphospecies using a stereomicroscope. Individuals belonging to two morphospecies from the genera *Culex* and *Wyeomyia* (dominant and ubiquitous filter-feeders in tank-bromeliads) were isolated in 20-mL test tubes and starved for 24 h. In order to obtain individuals of similar sizes and filtering abilities, all selected mosquitoes were at the third instar of larval development, corresponding to the average size of all of the individuals sampled. The experiment was carried out using 45-mL polypropylene tubes (Falcon®) at an ambient temperature (27°C throughout the experiment, close to the mean value of 25.3±1.0°C measured in the *C. berteroniana* tanks). Plastic tubes were used in four replicates and filled with a final water volume of 32.5 mL, as follows: (a) control with <150 µm water (with rotifers and without mosquito larvae), and (b) with mosquitoes, with <150 µm water containing rotifers and 10 additional mosquito larvae (two *Wyeomyia* sp. and eight *Culex* sp., based on percentages observed in our bromeliads) [26]. The density of the mosquito larvae (10 individuals in 32.5 mL of water) corresponded to the maximum density among the full range of natural densities generally found in tank-bromeliads [26] and is within the range of densities found in the American pitcher plant *S. purpurea* [27]. Samples were incubated for 24 h at an ambient temperature and fixed with 4 percent (final concentration) formaldehyde. Preservation and counting were carried out as described above for microorganisms. After being placed in the plankton chambers, the rotifers were counted using the inverted Leitz Laborlux microscope at ×100 magnification by scanning the entire chamber. Prior to fixing the samples, mosquito larvae were collected and fixed with alcohol (10%) to confirm the taxonomic identifications in the laboratory. Additional samples were collected at t = 0 in the <150 µm filtrates and used to estimate changes in the densities of the microorganisms and rotifers in control and treatment tubes over the 24-h experiment. Taxon-specific growth rates (μ, /d) in each experimental tube were calculated from changes in the numbers of cells assuming exponential growth according to the equation: μ = (ln*N_t_*−ln*N_0_*)/*t*, where μ is the apparent rate of population growth (/d), *N_0_* and *N_t_* are the initial and final abundances, and *t* is the duration of incubation. Grazing losses were estimated as the difference in growth rates between control and treatment tubes with larvae for each community.

### Data analysis

Because of unequal sample sizes and the lack of normality, the non-parametric Kruskal-Wallis test was used followed by a Mann-Whitney pairwise comparison to test differences in abiotic and biotic parameters between bromeliad species. Pearson's correlation coefficient was calculated to investigate relationships between algal abundance or biomass and abiotic and biotic parameters. Growth rate differences for microorganisms and rotifers between control and mosquito-treated tubes were tested using a one-way ANOVA. All statistical analyses were conducted using SYSTAT software (Systat Software Inc.; San Jose, CA, USA).

## Results

### Environmental conditions in the tanks of different bromeliads

Differences in reservoir height and diameter, and environmental conditions between plant species are provided in Table 1. A Kruskal-Wallis test indicated significant differences in both the height and the diameter of the bromeliad reservoir demonstrating the diverse morphological traits of the plants under study. Light intensity expressed as percentage of transmitted light was significantly different between sampling stations (Kruskal-Wallis test, *P*<0.001) and was 3 to 4 times higher at station #4 (an open area of the inselberg) than at the other stations (Mann-Whitney pairwise comparisons, *P*<0.01). Station #2 (tree gap) showed greater light intensity than stations #1 and #3 (Mann-Whitney pairwise comparisons, *P*<0.05). The water in the tanks was always slightly acidic, with extreme pH values ranging from 4.6 (*V. pleiosticha*) to 6.8 (*C. berteroniana*). Differences between plants were only significant for the central pools (Kruskal-Wallis test, *P*<0.05; Table 1). The minimum and maximum temperatures were recorded for *V. splendens* (22.8°C) and *G. lingulata* (26.8°C), respectively. Differences in temperature values between plants were significant both for the central pools and for the outer axils (Kruskal-Wallis test, *P*<0.05; Table 1). The values for both pH and temperature were not significantly different (Mann-Whitney pairwise comparisons, *P*>0.05) between the outer axils and the central pool within each bromeliad species.

### Abundance and community structure of the algae inhabiting tank-bromeliads

**Figure 3:**
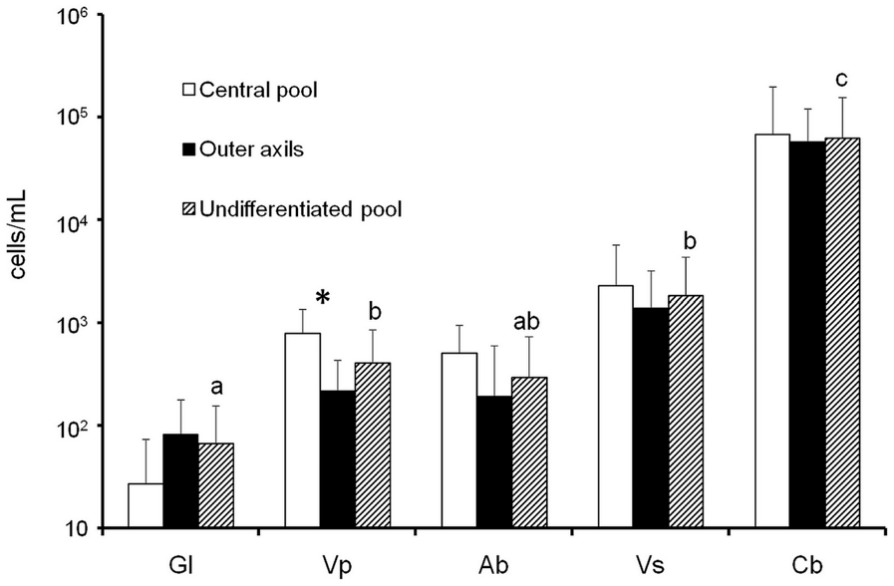
Mean abundances of algae according to the position of the foliar chamber (black and white bars) or regardless of the position of the foliar chamber (hatch marks). Hatch marks with the same letter indicate no significant difference (Mann-Whitney pairwise comparisons, *P*>0.05) between species. Black and white bars marked with an asterisk indicate significant within-species differences between the central pool and the outer axils (Mann-Whitney pairwise comparisons, *P*<0.05). Gl = *Guzmania lingulata*, Vp = *Vriesea pleiosticha*, Ab = *Aechmea bromeliifolia*, Vs = *Vriesea splendens*, and Cb = *Catopsis berteroniana*.

Algae were found in all five species of tank-bromeliads and in 71 percent of the samples. The mean density calculated from all of the samples was 9.5±40.5×10^3^ cells/mL with a maximum of 2.6×10^5^ cells/mL for *C. berteroniana*. Algal densities were significantly different (Kruskal-Wallis test, *P*<0.001) between plant species. Algal density for each plant species averaged between 66.4±88.3 cells/mL (*G. lingulata*) and 6.2±9.4×10^4^ cells/mL (*C. berteroniana*) and increased between the transitional forest and the open area of the inselberg (Fig. 3). Values were significantly higher in the tanks of *C. berteroniana* than in the tanks of all of the other species (*G. lingulata*, *V. pleiosticha*, *A. bromeliifolia* and *V. splendens*; Mann-Whitney pairwise comparisons: *P*<0.001, *P*<0.001, *P*<0.001 and *P*<0.05, respectively; Fig. 3, hatch marks). No significant difference in algal density was observed between *V. pleiosticha*, *A. bromeliifolia* and *V. splendens*, but the small and shaded bromeliad *G. lingulata* had a lower density of algae than *V. pleiosticha* and *V. splendens* (Mann-Whitney pairwise comparisons; *P*<0.05 and *P*<0.01, respectively). Significant differences in algal densities within species were only found for *V. pleiosticha*, with higher values in the central pools than in the outer axils (Mann-Whitney pairwise comparisons, *P*<0.01; Fig. 3, black and white bars). By pooling data from all species, the algal density was significantly higher in the central pools than in the outer axils (Mann-Whitney pairwise comparisons, *P* = 0.04; data not shown). In addition, algal density increased significantly with the intensity of transmitted light (Pearson's correlation coefficient, *R* ^2^ = 0.34, *P*<0.001) and bacterial abundances (*R* ^2^ = 0.20, *P*<0.001).

The algal community was represented by a handful of taxa, with *Bumilleriopsis* sp. (Xanthophyceae; Fig. S1) representing 92.5 percent and 78.5 percent of the algal abundance for *C. berteroniana* and *V. splendens*, respectively. This taxon was not found in other bromeliad species. *Euglena* sp. (Euglenophyceae) was present in the tanks of *V. pleiosticha*, *A. bromeliifolia* and *V. splendens*, accounting for up to 60 percent of the algal abundance. Other taxa were typically *Ankistrodesmus*-like cells (Chlorophyceae) and small (5 to 10 µm in diameter), unidentified spherical cells.

### Importance of algae and other microorganisms to microbial biomass

**Figure 4:**
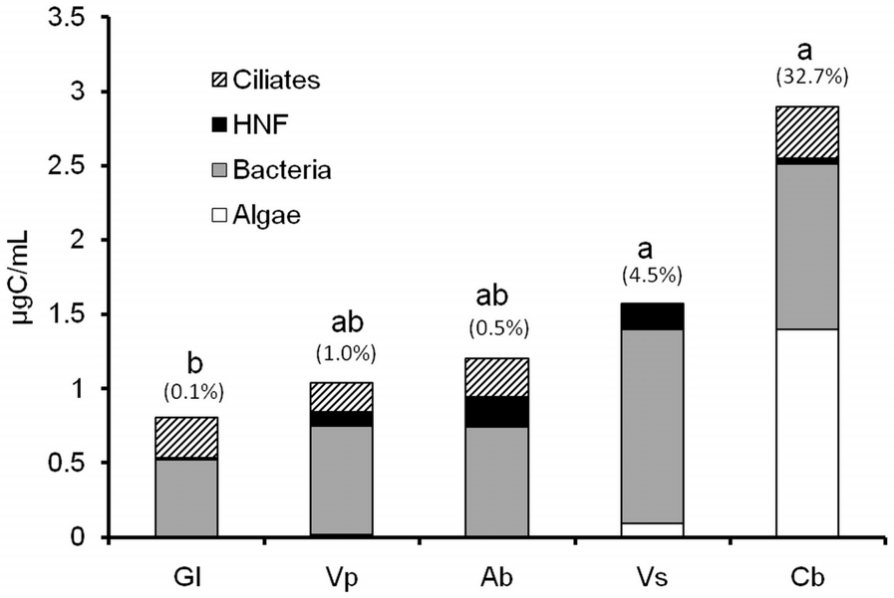
Mean biomass of microbial communities in five species of tank-bromeliads. Bars marked with the same letter indicate no significant difference in total biomass (Mann-Whitney pairwise comparisons, *P*>0.05). Values in parentheses indicate the mean relative contribution (%) of algae to total microbial biomass. Gl = *Guzmania lingulata*, Vp = *Vriesea pleiosticha*, Ab = *Aechmea bromeliifolia*, Vs = *Vriesea splendens*, and Cb = *Catopsis berteroniana*.

The mean biomass of the complete microbial community ranged from 0.8±0.4 µgC/mL (*G. lingulata*) to 2.9±3.0 µgC/mL (*C. berteroniana*) (Fig. 4). Although not significantly different between species (Kruskal-Wallis test, *P*>0.05), the microbial biomass was three times higher for *C. berteroniana* (*P*<0.05) as well as for *V. splendens* than for *G. lingulata* (Mann-Whitney pairwise comparisons, *P*<0.05) (Fig. 4). The microbial communities in all of the bromeliads investigated was dominated by heterotrophic bacteria (mean biomass = 0.8±0.7 µgC/mL) representing between 52.9 percent (*C. berteroniana*) and 85.3 percent (*V. splendens*) of the total microbial biomass.

The algal carbon content averaged 0.22±0.94 µgC/mL (maximal value: 6.04 µgC/mL in a central pool of an individual of *C. berteroniana*) and significant differences between species were observed (Kruskal-Wallis test, *P*<0.001). Values were higher for *C. berteroniana* (mean = 1.40±2.22 µgC/mL) than for all of the other bromeliad species (Mann-Whitney pairwise comparisons, *P*<0.05). Depending on the plant species, algae represented on average from 0.1 percent to 32.7 percent of the carbon content of the microbial communities (Fig. 4).

HNF biomass varied significantly between bromeliad species (Kruskal-Wallis test, *P*<0.001). The values were much lower for *G. lingulata* (mean = 0.01±0.01 µgC/mL) in comparison to *V. pleiosticha* (mean = 0.09±0.10 µgC/mL) and *A. bromeliifolia* (mean = 0.20±0.21 µgC/mL) (Mann-Whitney pairwise comparisons, *P*<0.001). Variations in the ciliate biomass (mean = 0.23±0.38 µgC/mL) between bromeliads was also significant (Kruskal-Wallis test, *P*<0.05). The carbon content of ciliates was lower for *V. splendens* (mean = 0.01±0.03 µgC/mL) than for *G. lingulata* (mean = 0.27±0.17 µgC/mL) and *V. pleiosticha* (mean = 0.19±0.27 µgC/mL), and slightly higher for *G. lingulata* than for *A. bromeliifolia* (mean = 0.26±0.53 µgC/mL). Microbial consumers (HNF and ciliates) averaged from 10.2 percent (*V. splendens*) to 39.5 percent (*A. bromeliifolia*) of the total microbial biomass according to plant species. Their contribution estimated from all of the samples regardless of plant species averaged 30.5%. Additional data (abundances of aquatic communities for each bromeliad species) are presented in Table S1.

### Impact of mosquito larvae on aquatic communities

**Figure 5:**
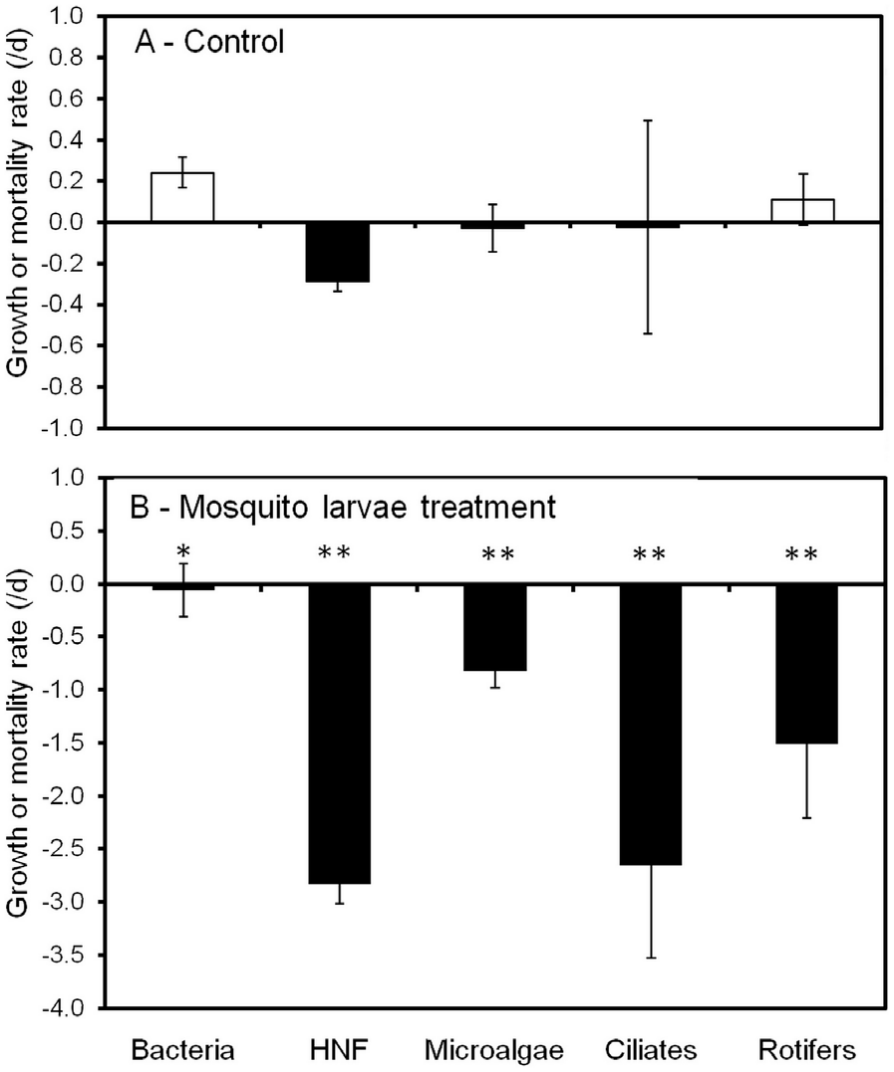
Growth (white bars) or mortality (black bars) rates of microorganisms determined from changes in densities during the 24-h experiment. Treatments are: (**A**) control and (**B**) with mosquito larvae. Values are the means of four replicates. Asterisks in (**B**) indicate significant differences in the values relative to (**A**) (One way ANOVA, *P<0.05, **P<0.01).

The growth rates of the aquatic communities in the control and treatment are shown in Fig. 5. Bacteria and rotifers showed a weak positive growth in the control. In contrast, the abundance of HNF decreased slightly during the 24-hr experiment, leading to a mean mortality rate of −0.2/d, while algae and ciliates showed near-zero growth in the control (Fig. 5A). All of the microbial communities and rotifers were significantly reduced by mosquito larvae treatments. Mortality rates in the mosquito treatment averaged from −0.1/d (bacteria) to −2.6/d (HNF) (Fig. 5B). Grazing losses, calculated as the difference between the treatment and control, fluctuated from −0.3/d to −2.6/d with higher values for protozoa and rotifers (−2.6, −2.5 and −1.5/d for ciliates, HNF and rotifers, respectively) compared to algae (−0.8/d) and bacteria (−0.3/d).

## Discussion

All of the five bromeliad species sampled at four dissimilar sampling stations contained algae. Algae were found in small numbers and in only 45.5 percent of the tanks for *G. lingulata*, although this species was located in the shaded forest and received only 15.8 percent of the transmitted light. In contrast, 100 percent of the tanks of *C. berteroniana*, an insectivorous bromeliad located at a highly exposed sampling station, always contained about 10^4^ algal cells per milliliter of water. These values are of the same order of magnitude as those reported for highly-productive freshwater pelagic environments [28], [29]. Thus, algae are abundant and omnipresent dwellers in this insectivorous tank-bromeliad. Light intensity clearly stimulated their growth as revealed by the positive correlation between algal abundances and the percentage of transmitted light. Moreover, while bacterial biomass largely dominated the biomass of the microbial communities in shaded bromeliads, the biomass of the primary producers was equivalent to the bacterial biomass in exposed plants. Algae accounted for, on average, one third of the total microbial biomass highlighting their potential roles as primary producers and sources of nutrient for *C. berteroniana*. We also show that, regardless of the plant species, the central pool promotes algal growth, probably as it is better placed than the outer chambers to receive nutrients and light [17]. Our assumption is that algal growth in shaded bromeliads could increase if sufficient light becomes available. The emergence of gap disturbances and the consequent lower input of leaf litter might thus enhance the autochthonous production of algae. This assertion is supported by the fact that the algal abundance increases as we move from the shaded forest species (*G. lingulata*) to bromeliads in the less-dense forest (*V. pleiosticha*, *A. bromeliifolia*, *V. splendens*), and to the species situated in an open area of the inselberg (*C. berteroniana*). It is also noteworthy that *Euglena* sp. represented a main part of the algal community in the bromeliads situated at the tree gap station and is known to have heterotrophic abilities [30] allowing growth to occur in environments with changing light conditions [31].

We expected the pH to increase with algal biomass as active photosynthesis results in the removal of free CO_2_, consequently increasing pH values in freshwater ecosystems [28], [29]. Accordingly, the highest mean pH value was found in the heliophilous bromeliad *C. berteroniana* which contained the highest algal abundances. Nevertheless, pH values were mostly slightly acidic and not significantly related to algal biomass. Laessle [17] recorded values ranging from 4.5 to 6.8 in a specimen of *Aechmea paniculigera* containing a large population of the green algae *Trachelomonas*. Similar values were found in bromeliads and in the pitcher plant *S. purpurea* where there were no autotrophic organisms [32], [33]. Water from the pitchers of allochtonous populations of *S. purpurea* containing large algal communities was also acidic [18], highlighting our finding of the minor impact of algae on pH values in the phytotelmatic ecosystem.

Algae contribute only a tiny part of the carbon in comparison to the detrital contents of the phytotelm. According to Richardson *et al*. [34], the quantity of fine particles (<1.5 mm) in the tanks of bromeliads situated in a dwarf forest represents 525.7 mg of carbon per plant. Even though *Catopsis berteroniana* is located in an open area in our study - which may limit the amount of intercepted litter, we assumed that there was an equivalent input of organic matter and as well as a similar size to those analyzed by Richardson *et al*. [34]. With this in mind, we estimated that algae constitute less than 0.02 percent of the organic detritus. This rough estimate shows that detrital processing from allochtonous organic matter remains the base of the food web in tank-bromeliads [7], [34], [35]. Nevertheless, in nature, algal production might still be high even without apparent elevated biomass values [36]. Owing to their small size, they are edible for microbial organisms as well as for invertebrates. In comparison with the entire litter content in the tanks of the bromeliads studied by Richardson *et al*. [34], invertebrates (which play a critical role in reducing the leaf litter) represented only 0.26 percent of the dry weight biomass. Such estimates indicate that living organisms represent a tiny part of the total amount of organic carbon in tank-bromeliads. Those comparisons may therefore fail to accurately evaluate the potential functional role of aquatic organisms, especially microorganisms which have high growth rates. Furthermore, algae are known to produce exudates, especially polysaccharides [37], and may provide key nutrients such as polyunsaturated fatty acids and sterols to higher trophic levels [38]. Algae undoubtedly represent a higher quality trophic resource than highly lignaceous, canopy-derived debris in phytotelmata, and could thus be more significant to the food web than their relative biomass would suggest [39].

Moreover, algal densities in this study were significantly related to bacterial densities, suggesting a positive interaction between the two functional communities. Such a relationship has not previously been observed between algae and bacteria in the tanks of an allochtonous population of *Sarracenia purpurea* [18]. Among-system correlations between bacteria and algae densities are well known in marine and freshwater pelagic environments [40], [41] where bacteria depend largely on the organic matter produced by phytoplankton. This commensalistic relationship [42], [43] appears in ecosystems where algae are the main carbon source. It is unlikely that bacteria grow exclusively on the exudates from algae in bromeliads and other phytotelmata, since inputs of detritus appear as the main carbon source for the food web. *Catopsis berteroniana* has been described as a protocarnivorous bromeliad [44] because not all of the characteristic features of a true carnivore, such as the synthesis of digestive enzymes, are present. In such a plant, it is obvious that animal detritus from trapped insects provides more highly nutrient-rich resources than materials from the leaf-litter [45], [46]. Thus, the decomposition of the prey residuals of entrapped insects may favour algal production which may in turn support a part of the bacterial production. Finally, without leaf-litter input in exposed environments, insectivorous bromeliads would provide algae with a valuable ecological niche.

The results of the experiment with aquatic communities of *C. berteroniana* suggested that the mortality of HNF in the control group stemmed from the grazing of rotifers leading to bacterial growth. The densities of algae and ciliates remained stable during the period, suggesting that these communities were relatively unaffected by the increase in bacterial cells or the abundance of rotifers during the course of the experiment. Bdelloids (the dominant rotifer group during this study; data not shown) are potential consumers of protozoa [27] and algae [47], and POM may constitute an important part of their diet [12], [33]. Our findings together with studies on communities inhabiting *S. purpurea* [11], [13], [14], [27], [48] show that the feeding activity of mosquito larvae greatly alters the prey community structure. A comparison of grazing losses between communities showed that protozoa and rotifers were the most heavily affected by predation by these invertebrates. Grazing loss estimates for bacteria and algae were less significant, though about twice as much for the latter. Lower grazing impact is probably due to the smaller size and higher turnover rates of these microorganisms, especially bacteria. In addition, *Bumilleriopsis* sp. is doubtless difficult to ingest because it forms filaments or radiating clusters (See Fig. S1) reducing grazing by filterers. Our estimation of an individual filtration rate of about 100 µL/ind/h for larvae is within the range (from 33 to 690 µL/ind/h) determined by Aly [49] from latex microspheres and yeast cells for Culicidae. The abundant mosquito larvae used in our experiment were among the highest abundances reported in field observations for *C. berteroniana* [26] and were generally higher than densities used in comparative experiments [11], [12]. In addition, the impact of mosquito larvae in the field is probably lower due to the presence of spatiotemporal microrefugia and habitat heterogeneity provided by natural bromeliads [50]. Nevertheless, our study clearly demonstrates that algae from the fluid of an insectivorous tank-bromeliad may significantly contribute to the diet of filterer mosquito larvae. This should encourage further field studies examining various bromeliad species from open habitats in order to evaluate the origin of the main sources of energy for aquatic food webs in bromeliads.

We conclude that algae are able to grow in the detritus-based food web of tank-bromeliads situated in a primary rainforest in the eastern Amazon. Algae were present in different species of plants sampled from diverse sites in the forest. Highest occurrence and densities were found in the tanks of the exposed bromeliad *C. berteroniana*. In this insectivorous plant, algae represent a significant share of the carbon in microbial communities and in the diet of mosquitoes. Therefore, algae might form the basis of a non-detrital food web, increasing the complexity of the food chain in exposed tank-bromeliads. Owing to the species richness of bromeliads, their diverse nutritional modes, and the large range of environmental conditions in which they grow in the Neotropical rainforest [1], the role of algae within the aquatic food web of tank-bromeliads deserves further investigation.

## Supporting Information

Figure S1 The algae *Bumilleriopsis* sp. under light microscopy (**A**) and epifluorescence microscopy (**B**) found in the tanks of *Vriesea splendens* and *Catopsis berteroniana* located on the inselberg of the Nouragues Research Station, French Guiana. Red color in (**B**) is due to the autofluorescence of chlorophyll *a* content under blue light excitation. Bars represent 5 µm.
(TIF)

Table S1 Mean abundances (± SD) of aquatic microbial communities in five species of tank-bromeliads situated in the Neotropical primary rainforest around the Nouragues Research Station, French Guiana.
(DOC)
