# Supplementary figures and images for: Are Algae Relevant to the Detritus-Based Food Web in Tank-Bromeliads?
Source: PLoS One. 2011 May 18;6(5):e20129. doi: 10.1371/journal.pone.0020129 (PMC3097239; doi:10.1371/journal.pone.0020129)

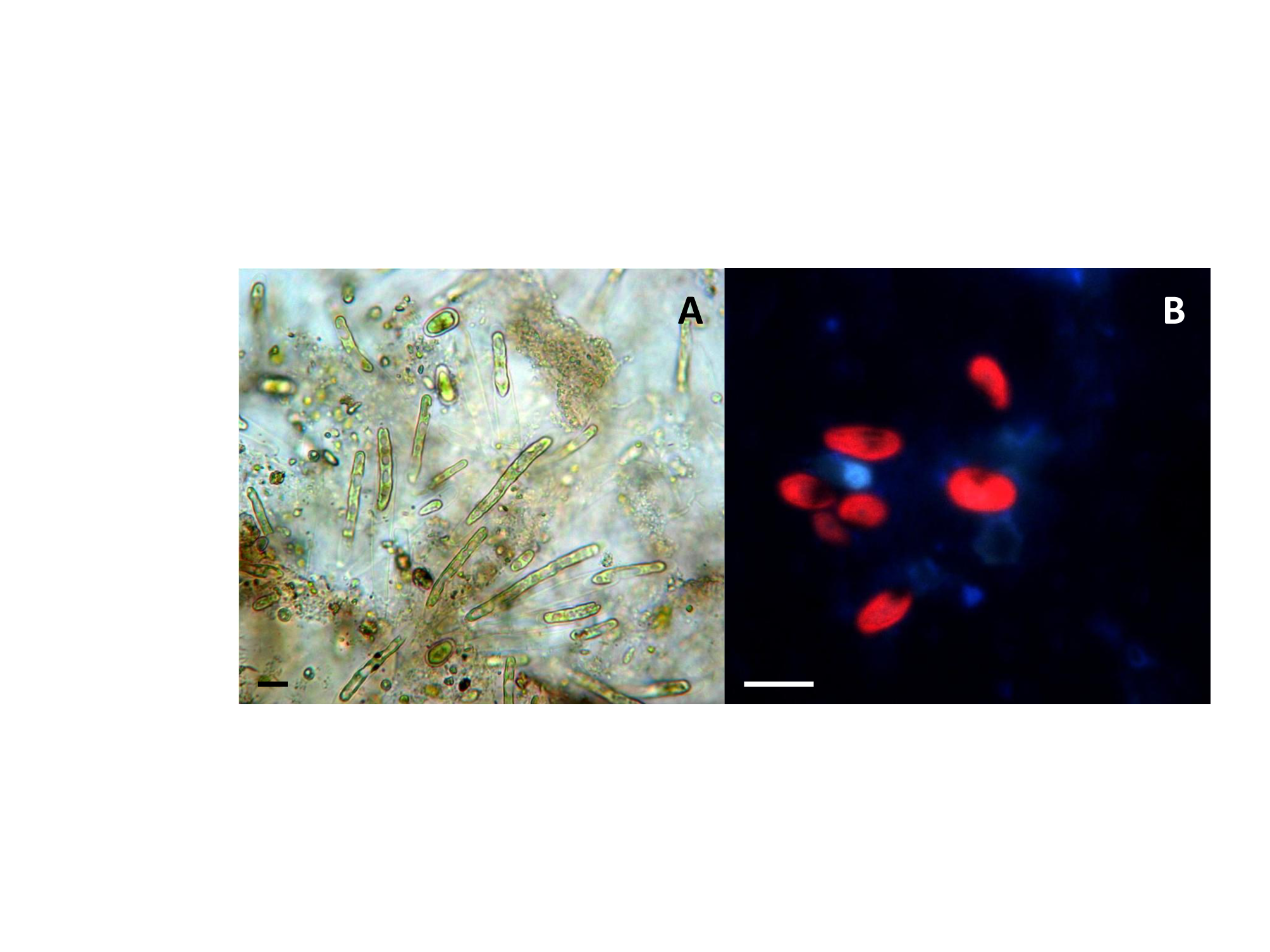

Supplement: Figure S1 — The algae Bumilleriopsis sp. under light microscopy (A) and epifluorescence microscopy (B) found in the tanks of Vriesea splendens and Catopsis berteroniana located on the inselberg of the Nouragues Research Station, French Guiana. Red color in (B) is due to the autofluorescence of chlorophyll a content under blue light excitation. Bars represent 5 µm. (TIF) [file pone.0020129.s001.tif]
