# Supplementary material for: Are Algae Relevant to the Detritus-Based Food Web in Tank-Bromeliads?
Source: PLoS One. 2011 May 18;6(5):e20129. doi: 10.1371/journal.pone.0020129 (PMC3097239; doi:10.1371/journal.pone.0020129)
Supplement: Table S1 — Mean abundances (± SD) of aquatic microbial communities in five species of tank-bromeliads situated in the Neotropical primary rainforest around the Nouragues Research Station, French Guiana. (DOC) [file pone.0020129.s002.doc]

| Bromeliad species | *Guzmania lingulata* | *Vriesea pleiosticha* | *Aechmea bromeliifolia* | *Vriesea splendens* | *Catopsis berteroniana* **^δ^** |
| --- | --- | --- | --- | --- | --- |
| Bacteria  (x 10^6^ cells/mL) | 1.60 ± 0.99 | 2.25 ± 2.49 | 2.26 ± 1.78 | 4.04 ± 1.29 | 3.42 ± 2.53 |
| HNF  (x 10^3^ cells/mL) | 0.87 ± 0.63 | 4.17 ± 3.11 | 2.20 ± 1.80 | 3.80 ± 3.58 | 7.00 ± 5.81 |
| Algae  (x 10^3^ cells/mL) | 0.07 ± 0.09 | 0.41 ± 0.45 | 0.29 ± 0.44 | 1.81 ± 2.49 | 62.35 ± 94.43 |
| Ciliates  (cells/mL) | 89.02 ± 55.47 | 79.73 ± 111.42 | 104.47 ± 213.15 | 5.00 ± 8.37 | 342.74 ± 551.77 |

**^δ^** insectivorous taxon
